# Synthesis and Characterization of ZIF-67 Mixed Matrix Nanobiocatalysis for CO_2_ Adsorption Performance

**DOI:** 10.3389/fbioe.2022.891549

**Published:** 2022-09-05

**Authors:** Saira Saeed, Rashdia Bashir, Shafique Ur Rehman, M. Tariq Nazir, Zeid A. ALOthman, Ahmed Muteb Aljuwayid, Amin Abid, Ahmad Adnan

**Affiliations:** ^1^ Department of Chemistry, GC University Lahore, Lahore, Pakistan; ^2^ Division of Science and Technology, University of Education Lahore, Lahore, Pakistan; ^3^ Department of Chemistry, Faculty of Sciences, University of Central Punjab, Lahore, Pakistan; ^4^ University of South Wales, South Wales, NSW, Australia; ^5^ King Saud Medical City, Al Riyadh, Saudi Arabia; ^6^ King Saud University, Riyadh, Saudi Arabia; ^7^ University of Sahiwal, Sahiwal, Pakistan

**Keywords:** nanobiocatalysis, ZIF 67, CO_2_ emission, nanotechnology, adsorption

## Abstract

In this study, ZIF-67-based mixed matrix membrane was synthesized with a solution casting method using tetrahydrofuran as the solvent. The as-synthesized ZIF-67 was characterized using PXRD, TGA, ATR-FTIR, and BET analysis for the surface area measurements. The minimum 3 wt% loading of ZIF-67 was incorporated within a hydrophobic polymer to evaluate the CO_2_ adsorption performance of ZIF-67. The stability of ZIF-67 in pure water and inorganic solvents was investigated. The maximum CO_2_ adsorption of the ZIF-67 mixed-matrix membrane (MMM) was 0.5 mmol/g at 273 K, which is higher than that of the pure polymer. The fabricated ZIF-67-based mixed-matrix membrane showed higher CO_2_ capture even at lower MOF loading using THF. The current study highly recommends the combination of hydrophobic polysulfone and a water-stable ZIF-67 for CO_2_ capture from wet flue gases.

## Graphical Abstract

Flowsheet diagram for ZIF-67 membrane preparation.

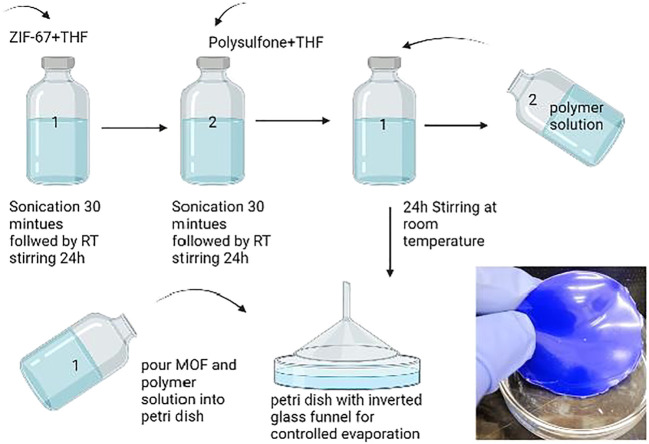

## Introduction

The burning of fossil fuels to meet the rising energy demand is the primary source of anthropogenic carbon dioxide (CO_2_) emissions in the atmosphere, which is a major contributor to greenhouse gas (GHG) emissions and global warming (Habib et al., 2020). The concentration of atmospheric CO_2_ has steadily increased during the last century, rising from 280 parts per million in the 1800s to more than 389 parts per million in 2019 and 400 parts per million in 2020 (Al-Rowaili et al., 2021). Adsorption processes based on porous solids (e.g., carbon materials) are becoming a great alternative to real technology based on absorption processes, utilizing basic solutions in terms of cost and environmental impact (Casco et al., 2014). Membrane-based gas separation techniques, on the other hand, provide an economically and technologically viable alternative for CCS (carbon, capture, and storage) due to their high energy efficiency, cheap capital cost, low carbon footprint, and simple and continuous operation mode (Cheng et al., 2019). In comparison to inorganic and molecular sieve materials, polymeric membranes have drawbacks such as lower polymer/particle interface compatibility. As a result, mixed-matrix membranes (MMMs) with a polymer matrix and an inorganic substance as the dispersion phase were developed. To improve polymer separation capabilities, various particles such as metal oxide nanoparticles and inorganic molecular sieves were introduced to the polymer matrix (Dorosti and Alizadehdakhel, 2018).

One of the most significant problems is that the fillers do not have adequate interfacial compatibility with the polymer matrix. MMMs frequently underperform their projected separation performance behavior due to poor adhesion between the polymer matrix and the fillers (Ishaq et al., 2019). Metal organic frameworks (MOFs) are made up of organic linkers, and the metal center functions as a filler in membrane preparation. MOFs have changeable pore/aperture size, high surface area, and specific adsorption affinity. As a result, MOF-based membranes have high potential for gas separation application (Yuan et al., 2017).

ZIFs (zeolitic imidazolate frameworks), a new type of metal organic frameworks (MOFs) with outstanding chemical and thermal stability, are being tested for a variety of applications such as gas adsorption and catalysis, etc. (Qian et al., 2012). The introduction of ZIFs into polymeric membranes improves the efficiency of gas separation processes. The Co-N bond in ZIF-67 is stronger than the Zn-N bond in ZIF-8, and the smaller pore size of ZIF-67 makes it a more suitable material for gas separation application (Gupta and Murthy, 2022). Zhang et al. (2012a) established successful separation output with C_3_H_6_-C_3_H_8_ by utilizing a zeolitic imidazolate framework. They found it excellent for C_3_H_6_/C_3_H_8_ separation due to effective pore size (3.4 A^o^) of ZIF-8 (Zhang et al., 2012b).

When evaluating MOFs for adsorption applications, their behavior in the presence of water is a critical consideration. Water vapor is present in many industrial streams and must be taken into consideration when choosing adsorbents for adsorption separation and purification systems (Burtch et al., 2014).

S. Meshkat et al. prepared a mixed matrix membrane using the solvent evaporation method with different loadings of ZIF-67 up to 5 wt. % with Pebax polymer for CO_2_/N_2_ gas separation application (Zhang et al., 2012b; Meshkat et al., 2020). N. Gupta et al. studied the effect of different wt.% loadings of ZIF-67 with polysulfone for extraction of NaNO_3_ and Na_2_SO_4_ from synthetic solutions using the phase inversion method (Gupta and Murthy, 2022). S. Feng et al. fabricated a mixed-matrix membrane incorporated with morphological regulated ZIF-67 nanosheets up to 5 wt.% loadings with Pebax using the solvent evaporation method for CO_2_/N_2_ separation application (Feng et al., 2020). Another scientific report suggested Pebax as a copolymer due to its thermoplastic elastomer property, with PA recognized for mechanical strengthening and PE for flexible gas transport (Barbi et al., 2003; Car et al., 2008). Hägg et al. (Nafisi and Hägg, 2014) reported that the permeability of CH_4_, N_2_, and CO_2_ was elevated by using ZIF-8 nanoparticles in Pebax polymer and fabricated dual sheet MMM. Furthermore, the selectivity parameter for CO_2_/NO_2_ decreased from 33.8 to 32.3 of the Pebax membrane in comparison with ZIF-8 loaded MMMs. Another study adapted IL@ZIF-8 socked with activated filler in methanol and BMIIm, finally blended with Pebax polymer. The 15 wt% MMM can efficiently raise the selectivity of CO_2_-CH_4_ and CO_2_-N_2_ by 92 % and 74%, respectively, whereas CO_2_ permeability by 45% (Li et al., 2016). Rodrigue et al. fabricated Pebax-based ZIF-67 and reported a 130% (162 barrer) increase in CO_2_ permeability. Another investigation on the fabricated ZIF-67 NS/Pebax reported enhanced CO_2_-N_2_ selectivity to 73.2 barrer and permeability of CO_2_ to 139.4 barrer, whereas DnBMCl Pebax 1,657 with ZIF8 association delivered high selectivities and permeabilities for CO_2_/CH_4_, CO_2_/H_2_, and CO_2_/N_2_ (Jomekian et al., 2017; Meshkat et al., 2020).

In this study, as far as our knowledge, there are no studies on ZIF- 67 based MMM using THF as a solvent. In this work, we synthesized a mixed-matrix membrane based on water stable ZIF-67 and hydrophobic polymer using the solvent evaporation method to check the CO_2_ adsorption performance of lower MOFs. In this work, ZIF-67 was made at room temperature, and a ZIF-67-based mixed matrix membrane was developed. The prepared ZIF-67 mixed-matrix membrane is highly stable after exposure to organic solvents and pure water. The CO_2_ adsorption isotherms were measured to evaluate the performance prepared membrane.

## Materials

Polysulfone (PS) average M_w_ ∼35,000 g/mol, density (1.24 g/ml at 25°C) by Sigma Aldrich and cobalt nitratehexahydrate (Co(NO_3_)_2_∙6H_2_O, 98%),2-methylimidazole (C_4_H_6_N_2_, 99%) were obtained from Sigma Aldrich. All solvents, such as methanol (CH_3_OH) and tetrahydrofuran (C_4_H_8_O), were analytical grade. All chemicals and glassy polymer were used without any processing. Pure gas CO_2_ was used for gas sorption analyses.

## Synthesis of ZIF-67

In a typical synthesis of ZIF-67 with some changes in the previously reported method, 1.436 g of Co (NO_3_)_2_·6H_2_O and 3.244 g 2-MeIm were weighed and separately dissolved in 100 ml of methanol. The metal solution was slowly poured into ligand solution under stirring at room temperature until the solution turned into dark purple in color. Then, stirring was carried out at room temperature for 3 h and the solution was allowed to stand without stirring at room temperature for 24 h. Afterward, the product was collected using centrifugation at 5000 rpm for 15 min. The washing was carried out three times with methanol and one time with chloroform to remove the dissolved impurities. The product was dried at 120°C in a vacuum oven for 3 days (Gupta and Murthy, 2022).

## Membrane Preparation

For the preparation of 3% ZIF-67 mixed-matrix membrane, 0.03 g of ZIF-67 was weighed in a 20-ml vial, and then 67 ml of THF was added into the vial. The solution was sonicated for 30 min followed by stirring for 24 h at room temperature. For polymer solution preparation, 0.97 g of polysulfone was taken in a 20-ml vial and then 67 ml of THF added to the vial, allowing it to stir for 24 h at room temperature. Then, the entire polymer solution was added into MOF solution and sonicated for 30 min followed by 24 h stirring at RT. After 24 h, sonication is carried out for 30 min before casting solution. The solution was poured into a glass petri dish and allowed to evaporate at room temperature under an inverted funnel. The inverted funnel was used for controlled evaporation of the solvent.

## Characterization Techniques

### Powder X-Ray Diffraction (PXR)

The XRD diffraction study of the sample was characterized by X-ray diffraction (Powder XRD, Bruker, D8). All the samples were analyzed with scan range 2θ from 3° to 45°, step size 0.02°, and time per step 0.2 s.

### Thermogravimetric Analysis (TGA)

The thermal resistance of all prepared samples was checked by using a thermo gravimetric analyzer TA Q500 (TA instruments, DE, United States). The samples were heated from 25°C to 590°C, while keeping a heating rate of 5°C/min under an N_2_ environment.

### Fourier Transform Infrared Spectroscopy (FT-IR) with Attenuated Total Reflectance (ATR)

The functional groups present in all fabricated MMMs were identified using an Agilent Cary 630 Fourier Transform Infrared Spectroscopy (FT-IR) with Attenuated Total Reflectance (ATR) crystal. All membrane samples were placed under the ATR-FTIR holder, and absorbance data were collected.

### Gas Sorption Analyses

The gas sorption analyses for CO_2_ (99.998%) were conducted on virgin Polysulfone, ZIF-67, and ZIF-67 membrane at 273 K using an Accelerated Surface Area & Porosimetry System (ASAP) 2,460 supplied by Micromeritics Instruments Inc. After cutting the membranes into small pieces, the fabricated membrane was activated by being subjected to vacuum at 70°C for 24 h. The N_2_ 77 K isotherm was used to evaluate the surface area (Langmuir and Brunauer–Emmett–Teller (BET) surface areas, etc.) of ZIF-67, while CO_2_ adsorption isotherms at 273 K were employed to evaluate the CO_2_ uptake performance of pure and mixed-matrix membrane based on ZIF-67.

## Results and Discussion

The results of different characterization techniques, that is, XRD, ATR -FTIR, TGA, and gas sorption analysis of ZIF-67-based mixed-matrix membrane are described below.

### Powder X-Ray Diffraction

XRD is a non-destructive technique. This technique is used to check the purity of the material, the impact of filler on polymer chain arrangement in the MMM, and the crystallinity of the MOF after incorporating it into the polymer.

**FIGURE 1:**
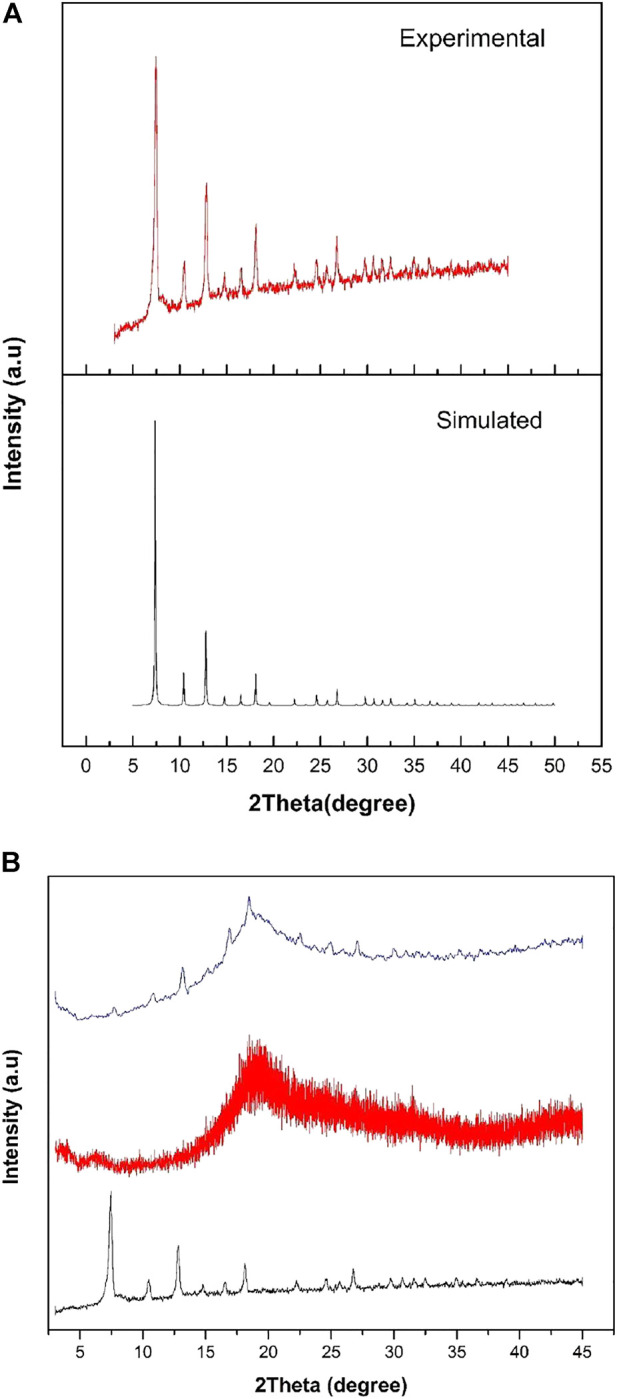
**(A)** PXRD pattern of simulated (Black) and experimental (red) ZIF-67 and **(B)** PXRD of ZIF-67(black), 3 wt% ZIF-67 MMM (blue) and pure polysulfone (red).

To find out the absence and presence of crystallinity of ZIF-67 in the MMM, XRD was performed in the 2θ range of 2°–45° Figure 1B. All diffraction peaks of as-synthesized ZIF-67 observed in Figure 1A matched well with their corresponding simulated pattern in literature (Kachhadiya and Murthy, 2021). This result indicates that the crystalline structure of ZIF-67 is well-maintained and no impurities are observed. The sharp and intense peak at 2θ position of 7.33° in Figure 1A proved the high crystalline nature of as-synthesized ZIF-67. The intensive peak in Figure 1A was observed at 2θ position of 7.33° attributed to (110) crystal plane, which is higher than other peaks (Mostafazadeh et al., 2018). In case of 3 wt.% ZIF-67 MMM, a very small peak is observed at 2θ position of 7.33ᵒ that confirms the presence of ZIF-67 in the membrane. However, the less intense peak observed in 3 wt.% MMM is due to the small wt.% content of ZIF-67 in polysulfone. The characteristic peak for polysulfone appeared in 2θ position of 18.49°.

### Thermal Gravimetric Analyses and Surface Area Measurement

**FIGURE 2:**
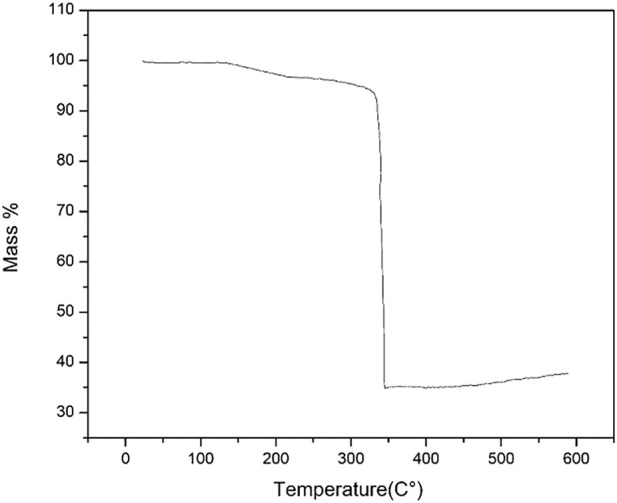
TGA of ZIF-67.

**FIGURE 3:**
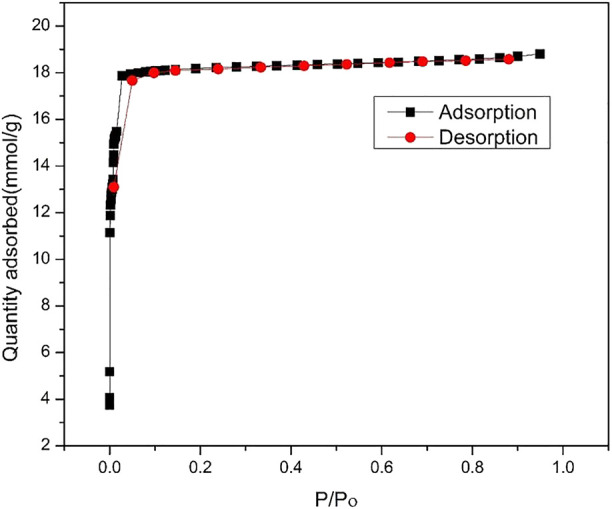
N_2_ 77K adsorption–desorption isotherm of ZIF-67.

The thermal stability of ZIF-67 was checked and shown in Figure 2 is 350°C with a minimum 3% mass loss that is in complete agreement with that of previously reported work. The initial mass percent loss is due to the removal of methanol. This stability of MOF is still considered better for MOF in terms of real membrane-based applications for gas separation (Qian et al., 2012). The N_2_ adsorption–desorption isotherm in Figure 3 reveals a reversible type 1 isotherm that is a characteristic of the microporous material. A plateau appears after certain uptake of gas at lower pressure, indicating that a multilayer is formed, and no more pores left for gas uptake. The sharp uptake of gas at higher pressure regions could be due to physiosorbed liquid N_2_. The BET area of as-synthesized ZIF-67 crystals is 1,696 m^2^/g higher than that of ZIF-67 synthesized using water as a solvent (Guo et al., 2016).

### Stability Testing

**FIGURE 4:**
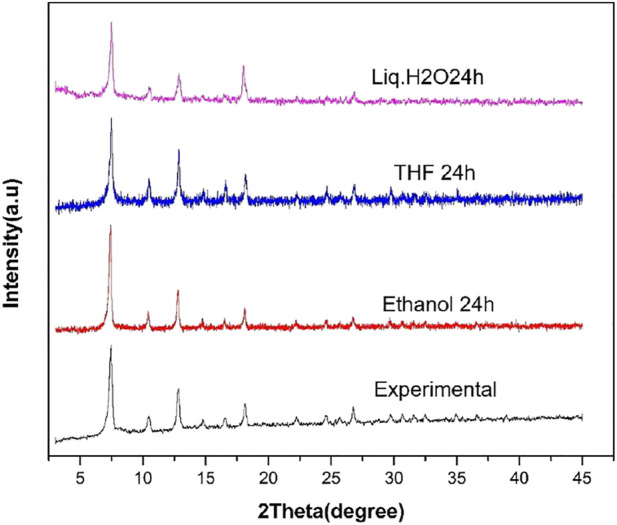
PXRD of ZIF-67 after soaking in ethanol (red), THF (blue), and liquid H_2_O (purple) for 24 h.

The water stability testing of ZIF-67 was carried out using different organic solvents and liquid water. The ZIF67 powder was immersed in ethanol, Liq. H_2_O, and THF for 24 h at room temperature. The PXRD pattern was checked after 24 h, as shown in Figure 4. It can be clearly observed from Figure 4 that there is no change in the crystal structure pattern of ZIF-67 after exposure to organic solvent and water. This result suggested that ZIF-67 is highly stable under pure water and organic solvents and can be utilized in future membrane-based applications for gas separation under humid conditions.

### ATR-FTIR

**FIGURE 5:**
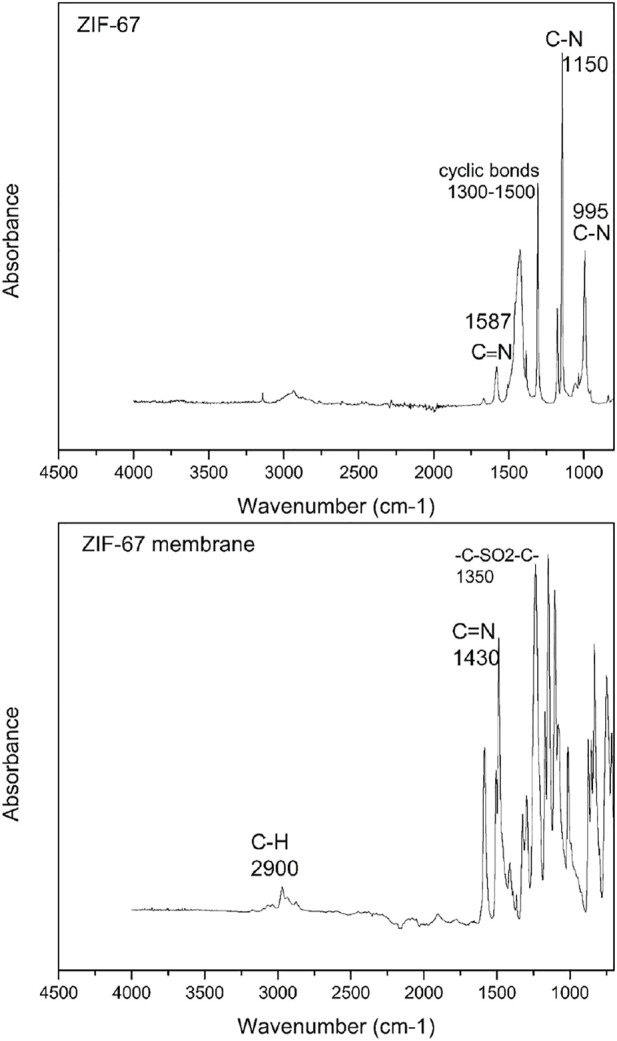
ATR-FT-IR of ZIF-67 and ZIF-67 membrane.

The ATR-FTIR spectra of ZIF-67- and ZIF-67-based mixed-matrix membrane in the wavenumber range of 700 cm^−1^ to 4500 cm^−1^are presented in Figure 5. The spectrum of as-synthesized ZIF-67 indicates the stretching and bending vibration of C-N at 995 cm^−1^ and 1150 cm^−1^. The bands observed at 3,000 cm^−1^ and 2900 cm^−1^ attribute the presence of aromatic and aliphatic C-H in the imidazole ring in case of ZIF67 (Yang et al., 2012). The incorporation of ZIF-67 within the polymer was successful, as shown in Fig (Dorosti and Alizadehdakhel, 2018). There were no new peaks observed in the ZIF-67 membrane which is the confirmation of no new chemical interaction occurred. The characteristic peak for polysulfone (-C -SO_2_-C-) was seen clearly at 1,350 cm^−1^ in the ZIF-67 membrane in Figure 5. The major peaks for 2 -methylimidazole (2-MeI) was observed in the ZIF-67 membrane in Figure 5 from 700 cm^−1^ to 1400 cm^−1^ due to the stretching and bending vibration of the imidazole functional group.

### Gas Sorption Analyses

**FIGURE 6:**
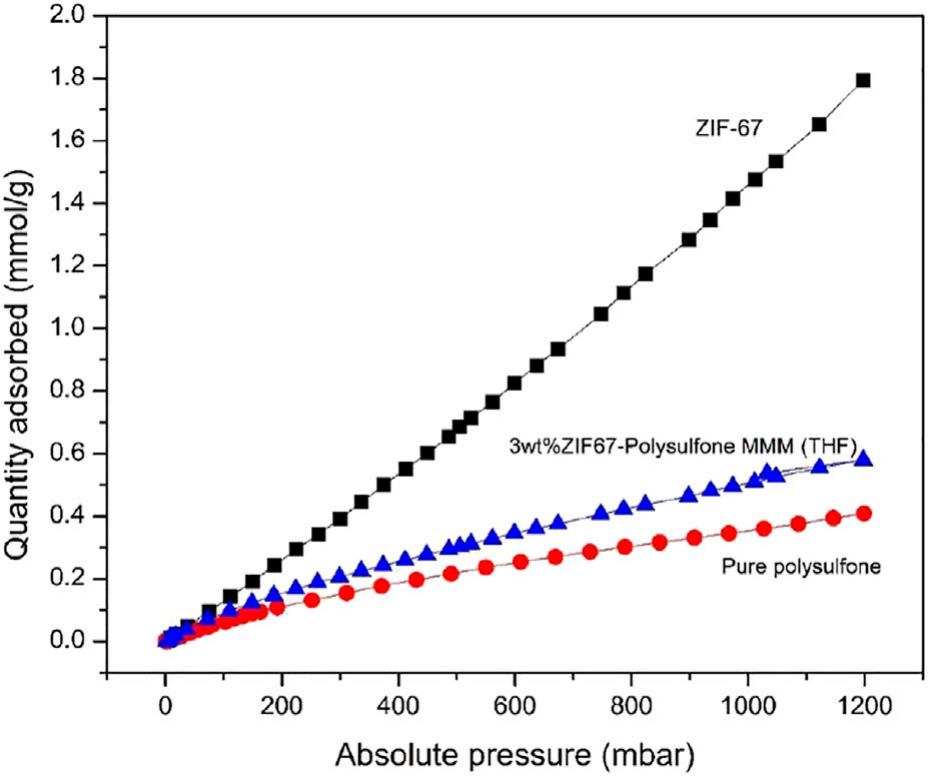
Gas adsorption isotherm of pure polysulfone (Red), ZIF-67 (black), and ZIF-67 MMM (blue) at 273 K.

The CO_2_ adsorption isotherm of pure polysulfone, ZIF-67, and 3 wt% ZIF-67-MMM was collected at temperature 273 K and pressure up to 1.2 bar to check the performance of lower MOF loading in polysulfone using THF as the solvent as shown in Figure 6. The maximum CO_2_ adsorption of ZIF-67 at 1bar is 1.47 mmol/g, which is in accordance with previously reported results (Yang et al., 2012), while CO_2_ uptake for 3 wt% ZIF-67-Polysulfone composite is 0.5 mmol/g higher than that of pure polysulfone uptake, that is, 0.3 mmol/g. The ZIF-67 maximum capacity for CO_2_ uptake was retained in the membrane even at minimum wt.% loading of MOF. This gas sorption results strongly recommend the use of ZIF-67 MMM as a competent candidate to capture CO_2_ from wet flue gases.

## Conclusion

In summary, a mixed-matrix membrane with lower MOF loading has been prepared with the solution casting method using THF as the solvent. The fabricated 3 wt.% ZIF-67 membrane showed higher CO_2_ uptake than the pure polymer. The prepared ZIF-67/polysulfonemixed matrix membrane was stable, flexible, and free standing. The as-synthesized ZIF-67 was highly stable in pure water and organic solvents. The combination of a hydrophobic polymer and a hydrophobic MOF was proved compatible with each other to fabricate a mixed-matrix membrane. This study provides an opportunity to utilize ZIF-67 MOF as competent material for CO_2_ capture from wet flue gases.

## Data Availability

The original contributions presented in the study are included in the article/Supplementary Material. Further inquiries can be directed to the corresponding author.
